# Intranasal Insulin Administration to Prevent Delayed Neurocognitive Recovery and Postoperative Neurocognitive Disorder: A Narrative Review

**DOI:** 10.3390/ijerph18052681

**Published:** 2021-03-07

**Authors:** Rafael Badenes, Ega Qeva, Giovanni Giordano, Nekane Romero-García, Federico Bilotta

**Affiliations:** 1 Department of Anesthesiology and Surgical Trauma Intensive Care, Hospital Clinic Universitari Valencia, University of Valencia, 46010 Valencia, Spain; nekaneromerog@gmail.com; 2 Department of Anesthesiology, Critical Care and Pain Medicine, Sapienza University of Rome, 00161 Rome, Italy; giordano.gj@gmail.com (G.G.); bilotta@tiscali.it (F.B.)

**Keywords:** intranasal insulin, postoperative cognitive dysfunction, neuroprotection

## Abstract

Delayed neurocognitive recovery and postoperative neurocognitive disorders are major complications of surgery, hospitalization, and anesthesia that are receiving increasing attention. Their incidence is reported to be 10–80% after cardiac surgery and 10–26% after non-cardiac surgery. Some of the risk factors include advanced age, level of education, history of diabetes mellitus, malnutrition, perioperative hyperglycemia, depth of anesthesia, blood pressure fluctuation during surgery, chronic respiratory diseases, etc. Scientific evidence suggests a causal association between anesthesia and delayed neurocognitive recovery or postoperative neurocognitive disorders, and various pathophysiological mechanisms have been proposed: mitochondrial dysfunction, neuroinflammation, increase in tau protein phosphorylation, accumulation of amyloid-β protein, etc. Insulin receptors in the central nervous system have a non-metabolic role and act through a neuromodulator-like action, while an interaction between anesthetics and central nervous system insulin receptors might contribute to anesthesia-induced delayed neurocognitive recovery or postoperative neurocognitive disorders. Acute or chronic intranasal insulin administration, which has no influence on the blood glucose concentration, appears to improve working memory, verbal fluency, attention, recognition of objects, etc., in animal models, cognitively healthy humans, and memory-impaired patients by restoring the insulin receptor signaling pathway, attenuating anesthesia-induced tau protein hyperphosphorylation, etc. The aim of this review is to report preclinical and clinical evidence of the implication of intranasal insulin for preventing changes in the brain molecular pattern and/or neurobehavioral impairment, which influence anesthesia-induced delayed neurocognitive recovery or postoperative neurocognitive disorders.

## 1. Introduction

The nomenclature of postoperative cognitive dysfunction (POCD) is not defined in the Diagnostic and Statistical Manual of Mental Disease (DSM-5), and this has been associated with limitations such as an inconsistent definition and consequently recognition by non-anesthesia specialists [1,2]. To facilitate broad recognition, new terminology aligned with that of DSM-5 was adopted. The clinical nomenclature was proposed to be changed from POCD to delayed neurocognitive recovery (DNR) for neurocognitive impairment identified from 7 to 30 days postoperatively and postoperative neurocognitive disorder (pNCD) from the 30th postoperative day to the 12th postoperative month [1].

DNR and pNCD are major complications of surgery, hospitalization, and anesthesia that are receiving increasing attention [3,4,5]. Their incidence depends on the type of surgery, cognitive performance tests, time of postoperative assessment, and specificity and sensibility of the cognitive tests [6]. It is reported to be approximately 11% after non-cardiac surgery and 60% in cardiac surgery patients [4]. This cognitive decline is associated with poorer recovery, increased use of social financial assistance, and a higher mortality rate [6,7].

Several preoperative, intraoperative, and postoperative risk factors are associated with DNR/pNCD, which include advanced age, education level, history of diabetes mellitus (DM), malnutrition, perioperative hyperglycemia, depth of anesthesia, blood pressure fluctuation during surgery, chronic respiratory diseases, etc. [5,8]. Despite the very limited evidence of a causative relationship between anesthetics and cognitive impairment >6 months after anesthesia, there is a lot of scientific evidence that suggests a causal association between anesthesia and DNR/pNCD within 6 months postoperatively, and various pathophysiological mechanisms have been proposed: mitochondrial dysfunction, neuroinflammation, calcium dysregulation, increase in tau protein phosphorylation, accumulation of amyloid-β (Aβ) protein, etc. [8,9,10,11,12]. Pharmacological and non-pharmacological approaches have been proposed to reduce or prevent the incidence of DNR/pNCD [8].

The central nervous system insulin receptors (CNS-IRs) have the highest concentration in the thalamus, caudate-putamen, hippocampus, amygdala and parahippocampal gyrus; intermediate concentration in the cerebellum, cerebral cortex, and caudate nucleus; and the lowest concentration in the substantia nigra, red nucleus, white matter, and cerebral peduncles [13,14]. Downregulation of insulin transport through the blood–brain barrier (BBB) and dysregulation of the insulin receptor (IR) intracellular cascade that occur in patients with DM are associated with a higher risk of Alzheimer’s disease (AD) because of Aβ peptide accumulation and increased neuronal tau protein phosphorylation [15]. The characteristic distribution of CNS-IRs and the proven association between IR dysregulation and chronic cognitive impairment (as reported in patients with AD) suggest a non-metabolic role of insulin in cognitive performance, memory, and neuromodulation [16]. This role can indicate that CNS-IRs act as neuromodulator-like mediators, and this might partially explain how the interaction between anesthetics and CNS-IRs might contribute to anesthesia-induced DNR/pNCD [13,17]. Perioperative intranasal insulin administration, possibly with a direct action on IRs, is proven to restore more effective signaling, thus preventing the onset of DNR/pNCD [18,19]. This narrative review was accomplished through a literature search of PubMed, EMBASE, and SCOPUS online medical databases, including only articles in English. The following search terms were used: anesthesia, delayed neurocognitive recovery, postoperative neurocognitive disorder, intranasal insulin, Alzheimer’s disease, and Parkinson’s disease.

The aim of this review is to report preclinical and clinical evidence of the implication of intranasal insulin for preventing changes in the brain molecular pattern and/or neurobehavioral impairment, which influence anesthesia-induced DNR/pNCD.

## 2. Preclinical Evidence

Several preclinical studies have addressed the therapeutic role of intranasal insulin in preventing post-anesthesia changes in the brain molecular pattern and/or neurobehavioral impairment in animal models (Table 1).

A study published in 2014 investigated the effects of intranasal insulin on anesthesia-induced hyperphosphorylation of tau protein in the brain tissue of 3xTg-AD mice, a commonly used transgenic model of AD [20]. Intranasal insulin or saline (as control) was administered daily in 3xTg-AD and wild-type mice for seven consecutive days. After seven days, anesthesia was induced with intraperitoneal propofol or intralipid (as a control). Brain tissue was then prepared for Western blot and immunohistochemical analyses. In mice anesthetized with propofol, there was a marked increase in the phosphorylation status of tau protein when compared to controls; moreover, daily intranasal insulin administration before anesthesia was found to be associated with significantly lower phosphorylation levels of tau protein when compared to saline. These findings represent the first evidence supporting that intranasal insulin might ameliorate anesthesia-induced DNR, pNCD, and AD-like brain histological changes.

In 2016 and in 2017, the same group published two studies on the efficacy of intranasal insulin in preventing anesthesia-induced spatial learning and memory deficit in mice [21,22]. The aim of the first one was to report whether pretreatment with intranasal insulin could prevent anesthesia-induced deficits in spatial learning, memory, and hyperphosphorylation of tau protein [21]. Intranasal insulin or saline (as the control) was administered daily in wild-type mice for seven consecutive days. On the day after the last dose was administered, anesthesia was induced with intraperitoneal propofol or intralipid (as the control), followed by 2.5% sevoflurane inhalation for 1 h. Mice were sacrificed at different times (immediately 24 h or 5 days post-anesthesia), and brain tissue was prepared for Western blot and immunohistochemical analyses, while the Morris water maze test was used to evaluate the mice’s spatial learning and memory. Anesthesia-treated mice showed higher phosphorylation levels of tau protein and impairment in spatial learning and memory when compared to non-anesthetized controls. Moreover, anesthesia-induced hyperphosphorylation of tau protein in the brain samples and impairment in spatial learning and memory were both prevented in mice treated with intranasal insulin administered before anesthesia when compared to controls. The study published in 2017 was aimed to test the effects of intranasal insulin administration and anesthetic exposure on neurobehavioral performance such as spatial learning and memory in mice of different ages and conditions [22]. Intranasal insulin was administered for three consecutive days and was followed by anesthesia induced with intraperitoneal propofol or intralipid (as a control) and maintained by inhalation of 2.5% sevoflurane for 1 or 3 h. Deficits in spatial learning and memory, as tested using the Morris water maze 2–6 days after anesthesia exposure, were identified in aged (17–18 months old) wild-type mice and in adult (7–8 months old) 3xTg-AD mice but not in adult wild-type mice. Long-term neurobehavioral changes were also reported in 3xTg-AD mice after anesthesia exposure. Intranasal insulin administration before anesthesia prevented deficits in spatial learning and memory and long-term neurobehavioral changes in 3xTg-AD mice. These findings suggest that aging and prior existence of AD-like brain pathology might be associated with higher vulnerability to anesthesia-induced DNR/pNCD onset and that intranasal administration of insulin can effectively prevent these disorders.

Based on this preliminary evidence, two randomized controlled preclinical trials published in 2019 and 2020 investigated the role of intranasal insulin administration in preventing neurobehavioral modifications during general anesthesia administration in neonatal mice [23,24]. In the study published in 2019, the authors’ aim was to address the long-term effects of general anesthesia with sevoflurane in a neonatal mice model and to study the role of intranasal insulin in preventing anesthesia-related brain damage and neurobehavioral modifications [23]. Seven-day-old neonatal mice were randomly assigned into four groups: non-anesthetized mice + saline (controls); non-anesthetized mice + insulin; anesthetized + saline; and anesthetized + insulin. Anesthesia was induced using 5% sevoflurane and maintained with 3% sevoflurane for 3 h for 3 consecutive days. In mice treated with intranasal insulin, 0.14 international units (IU) were delivered 30 min before each anesthesia procedure. Behavioral tests included an open-field test for assessing general spontaneous activity and anxiety, a novel object recognition test for assessing memory, the Morris water maze test for assessing hippocampal spatial learning, and a memory and fear conditioning test to assess learning and memory that involve the amygdala. According to the results provided by the authors, while general anesthesia was demonstrated to induce long-term behavioral abnormalities; promote changes in synaptic proteins, in particular post-synaptic density protein 95 (PSD95); and promote neuronal apoptosis in neonatal mice brains, intranasal insulin administration was found to prevent these abnormalities. In the randomized controlled preclinical trial published in 2020, the authors investigated the relationship between anesthesia exposure, neurobehavioral modifications, and intranasal insulin administration in neonatal mice [24]. Neonatal mice (7 days old) were randomized into four groups: non-anesthetized mice + saline (controls); non-anesthetized mice + insulin; anesthetized mice + saline; and anesthetized mice + insulin. Anesthesia was induced with 5% sevoflurane and maintained with 3% sevoflurane for 3 h for a total of 3 consecutive days. Before each anesthesia procedure, the neonatal mice received intranasal administration of insulin or saline. All mice were tested at different times, including at old age (18 months old), with the following behavioral tests: open field, novel object recognition, Morris water maze, and fear conditioning. Brain samples were prepared for Western blot analysis. The authors showed that compared to non-anesthetized controls, neonatal mice exposed to anesthesia were not affected in spontaneous activity or anxiety when evaluated at old age. However, neonatal mice exposed to anesthesia did develop long-term cognitive impairment, including learning and memory deficit associated with specific brain areas such as the amygdala, hippocampus, frontal cortex, and cingulate cortex. Molecular investigations of the brains of aged mice reported no detectable alterations in the levels of tau phosphorylation or synaptic proteins (including PSD95) when comparing anesthetized mice with controls. Therefore, intranasal insulin administered before anesthesia induction can prevent the development of anesthesia-related behavioral and cognitive impairment.

To investigate the molecular mechanisms involved in insulin-induced prevention of anesthesia-related cognitive impairment, several intra- and extracellular brain factors were measured in wild-type, aged mice exposed to anesthesia [25]. Mice were treated with intranasal insulin administration or saline (as a control) for 7 consecutive days. The day after the last administration, the mice received anesthesia (intraperitoneal injection of propofol for induction and sevoflurane inhalation for 1 h as maintenance) or intralipid as a control. A group of mice was sacrificed, a group was tested with the Morris water maze test for spatial learning and memory evaluation, and a group was tested with an inhibitor of eukaryotic elongation factor 2 kinase (eEF2K), a blocker of the mammalian target of rapamycin-eukaryotic elongation factor 2 (mTOR-eEF2) signaling pathway. In this study, anesthesia was proven to induce a reduction in brain synaptic proteins and brain-derived neurotrophic factor (BDNF), while prior administration of the eEF2K inhibitor or intranasal insulin administration promoted the mTOR-eEF2 signaling pathway and effectively prevented anesthesia-induced reduction in brain synaptic proteins and BDNF and neurocognitive disorders (NCD).

Intranasal insulin administration in aged mice was also proven to prevent anesthesia-induced abnormalities of glycogen synthase kinase 3 beta (GSK-3β) and phosphoinositide 3-kinase/pyruvate dehydrogenase kinase 1/protein kinase B (PI3K/PDK1/Akt) signaling pathways, expression of synaptic proteins, and neurobehavioral impairment [26]. GSK-3β is a protein kinase involved in tau hyperphosphorylation; PI3K/PDK1/AKT is involved in the insulin signaling pathway and plays a role in preventing GSK-3β-mediated tau hyperphosphorylation. Intranasal insulin or saline (as a control) was administered daily for 26 days. From day 7 and for 5 consecutive days, general anesthesia was induced and maintained for 2 h after intraperitoneal injection of propofol. From the 27th day, intranasal insulin administration or saline was continued for 15 days and neurobehavioral tests (such as object recognition test, fear-conditioning test) were administered. Intranasal insulin administration was shown to prevent NCD induced by anesthesia in aged mice when compared to controls. Moreover, insulin upregulated the PI3K/PDK1/AKT signaling pathway, attenuated the hyperphosphorylation of tau induced by GSK-3β, and prevented the loss of specific synaptic proteins (such as PSD95) involved in the signal pathway of cognition, memory, and movement.

## 3. Clinical Evidence

The hormone insulin was isolated from dogs for the first time in 1921 by Dr. Frederick Banting, a Canadian doctor surgeon, and Charles Best, a medical student [27] (Table 2). It was subsequently injected intravenously or subcutaneously into animal and human models. Clinical observations reported the following conclusions: reduction in the blood glucose concentration, abolishment of glycosuria, disappearance of acetone bodies from the urine, and increase in the use of carbohydrates. The use of insulin as non-glycemia management therapy dates back to the 1930s, when Manyfreed Sakel used it, with intravenous administration, to treat morphine addiction and schizophrenia [28]. Sakel’s method consisted in a four-phase approach that led to an insulin-induced coma: preparatory phase, shock phase, rest phase, and terminal phase. Patients with schizophrenia reported a reduction in or the disappearance of all sorts of hallucinations during insulin-induced hypoglycemia and a protraction of the lucid phase’, thus proving the clinical evidence of a psychotropic effect of insulin [28]. Furthermore, hypoglycemia shock induced by insulin was used in dementia praecox patients [29]. All these approaches were abandoned after the introduction of antipsychotic drugs (such as chlorpromazine) into clinical practice [30].

Plasma insulin arrives at the brain interstitial fluid and cerebrospinal fluid (CSF) via an IR-mediated transcytosis mechanism through endothelial BBB cells [31]. In addition, some brain areas, such as the hypothalamus, hippocampus, and brain stem, are shown to independently produce insulin [32]. Intranasal insulin administration in humans has been proven to be feasible, safe, effective, and independent of the BBB [33]. The administration through this route uses olfactory and trigeminal neurons that pass through the cribriform plate and induces rapid distribution to the CNS (within minutes) [33]. The peptide hormone is detectable in the CSF for at least 80 min, and less than 3% of the administered insulin reaches the systemic bloodstream without causing systemic hypoglycemia or hepatic first-pass metabolism [33].

Intranasal insulin administration was shown to have pleiotropic effects during acute, subacute, and chronic phases after acute ischemic stroke events [34]. During the acute phase, insulin suppresses the pro-inflammatory transcription response, induces vasodilatory effects by promoting activation of endothelial nitric oxide synthase, enhances the effects of thrombolysis, and reduces the final infarct volume. In addition to the acute phase, insulin’s effects extend to the subacute and chronic phases through an anti-apoptotic effect, promotion of neurite regeneration, neurotransmission, and functional connectivity [33,34]. Effects on neurocognitive and memory performance were positive according to the results obtained in 38 healthy individuals, without memory impairment, evaluated with a double-blind and between-subject comparison that showed improved word recall ability and self-confidence in cognitive tasks after 8-week treatment [35]. Another systematic review showed that only high doses of intranasal insulin (160 IU/die) compared to lower doses (≥60 IU/die) induced potential beneficial effects in healthy people, with greater improvements in females when compared to men [36].

Recent clinical evidence supports intranasal insulin administration also in memory-impaired patients such as those with mild cognitive impairment (MCI), AD, Parkinson’s disease (PD), and multiple system atrophy diagnosis [37,38,39,40,41,42,43]. The therapeutic effects of intranasal insulin administration in 26 memory-impaired individuals (13 with early AD and 13 with amnestic MCI) and 35 controls were evaluated [37]. Insulin treatment facilitated recall of verbal memory, with stronger effects in memory-impaired apolipoprotein E4 (APOE)– patients compared to APOE4+ ones. Another systematic review including seven studies and a total of 293 patients showed that intranasal insulin administration in patients with MCI or AD improved verbal memory and story recall, especially for APOE4– patients [38]. It is unclear whether the difference is due to the stronger association between insulin resistance and AD in patients without as compared to those with the risk allele or whether insulin administration aggravates impairments in brain glucose metabolism in carriers of the APOE4+ genotype [39]. Furthermore, there were positive results in functional status and daily activity. Daily intranasal insulin therapy for 4 months in patients with MCI and AD improved delayed memory and preserved the brain volume by reducing the progression of brain hypometabolism [40]. The role of intranasal insulin administration was investigated in two randomized controlled trials (RCTs) enrolling, respectively, 104 and 60 MCI or AD patients [18,41]. In the first one, insulin was administered for 4 months, while in the second one, long-lasting insulin detemir administration was conducted for 21 days. Insulin administration improved verbal, visuospatial, and working memory and preserved general cognition and functional abilities, while placebo-assigned participants showed decreased fludeoxyglucose 18 uptake in the parietotemporal, frontal, precuneus, and cuneus regions. A prospective, randomized, double-blinded, placebo-controlled, pilot study of 16 enrolled patients (15 with Parkinson’s disease and 1 with multiple system atrophy diagnosis) reported that intranasal insulin administration for a 4-week period improved cognitive and motor performance in PD patients, while there was a lack of disease progression in the multiple system atrophy case, compared to intranasal sterile saline administration [42].

## 4. Discussion

This narrative review is intended to report available preclinical and clinical evidence of the implication of intranasal insulin in preventing changes in the brain molecular pattern and/or neurobehavioral impairment, which influence anesthesia-induced DNR/pNCD.

**Figure 1:**
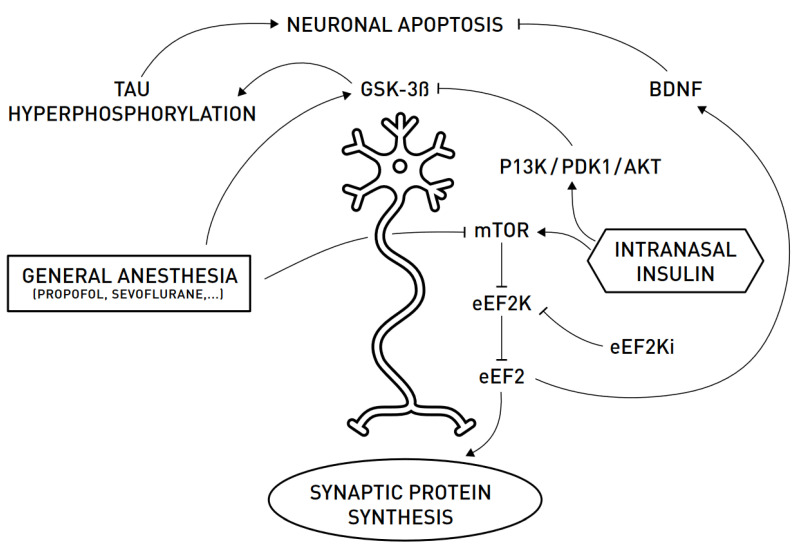
A schematic representation of the relationship between the neuron (in the center) and how insulin can possibly blunt anesthetic-induced apoptosis and tau phosphorylation. AKT, protein kinase B; BDNF, brain-derived neurotrophic factor; eEF2, eukaryotic elongation factor 2; eEF2K, eukaryotic elongation factor 2 kinase; eEF2Ki, eEF2K-inhibitor; GSK-3β, glycogen synthase kinase 3 beta; mTOR, mammalian target of rapamycin; PDK1, pyruvate dehydrogenase kinase 1; PI3K, phosphoinositide 3-kinase.

Collected preclinical evidence shows that anesthesia administration enhances the phosphorylation status of tau protein in the brain, reduces the expression of brain synaptic proteins and BDNF, and induces cognitive decline both in wild-type and AD models, including adult and aged mice; long-term neurobehavioral effects are also demonstrated when anesthesia is administered in neonatal mice. As suggested by preclinical evidence, insulin can blunt anesthetic-induced apoptosis and tau phosphorilation at various levels (Figure 1). While biochemical changes, including hyperphosphorylation of tau protein, are reported to be transient, long-lasting cognitive and neurobehavioral effects have been reported and confirmed by several studies. Intranasal administration of insulin has been found effective in preventing biochemical, cognitive, and neurobehavioral changes that are induced by anesthesia.

General anesthetics contribute to DNR/pNCD by indirectly promoting neuronal apoptosis and by interfering with synaptic protein synthesis. Neuronal apoptosis is favored by the hyperphosphorylation of tau protein mainly by the kinase GSK-3β, which is stimulated by anesthetics. Moreover, the inhibition of the mTOR-eEF2 pathway leads to a reduction in specific synaptic proteins and BDNF synthesis. Intranasal administration of insulin has been proven to reduce GSK-3β activity, through the activation of the PI3K/PDK1/AKT signaling pathway, and to stimulate the mTOR-eEF2 pathway, thus resulting in counteracting the deleterious effects of general anesthesia.

Insulin is a peptide hormone, and the blood glucose concentration is the principal regulator of its secretion [13]. IRs are found in many tissues in different concentrations and present an intracellular tyrosine phosphorylation transduction that defines two major insulin signaling pathways: (1) PI3K/PDK1/AKT, which promotes intracellular glucose transport, glycogen, protein, and lipid synthesis; stimulates axonal outgrowth; and has an anti-apoptotic pathway inhibiting proapoptotic proteins, and (2) mTOR/eEF2K/eEF2, which promotes mitosis by gene transcription, cell proliferation, survival, motility, and protein synthesis. There is some crosstalk between these two intracellular pathways. The CNS-IRs have a characteristic distribution in the brain, with the highest concentration in the thalamus, caudate-putamen, hippocampus, amygdala, and parahippocampal gyrus; intermediate concentration in the cerebellum, cerebral cortex, and caudate nucleus; and the lowest concentration in the substantia nigra, red nucleus, white matter, and cerebral peduncles. This specific distribution and the anti-apoptotic and cell proliferation action of the intracellular signaling pathway suggest that CNS-IR function may relate to cognitive performance, memory, and neuromodulation because of the effects of insulin on neuronal metabolism, neuronal function, and neurotransmission. Insulin exerts a trophic function in the CNS by regulating cell growth, differentiation, and neuronal survival. Furthermore, insulin has a neuromodulatory role as it participates in synaptic plasticity by modulating the activities of excitatory and inhibitory receptors.

Occurrence of DNR/pNCD is among the most serious adverse complications after surgery and anesthesia that cause poor recovery, increased use of social-financial assistance, and a higher mortality rate [7,43]. It is associated with memory and language impairment and might last for months or even years [9]. The pathogenesis is still unclear, but risk factors such as advanced age, low baseline cognition, education level, history of DM, dehydration, malnutrition, major surgery (cardiac and orthopedic), intraoperative blood pressure fluctuation and hyperglycemia, postoperative respiratory complications, type and depth of anesthesia, etc., have been shown to contribute [8]. Anesthesia was shown to evoke a systemic and neuroinflammatory response, accumulation of Aβ proteins, increase in tau protein phosphorylation, mitochondrial dysfunction, and calcium dysregulation [44].

To prevent this serious complication, several pharmacological and non-pharmacological strategies have been tested [8,43]. A systematic review tested 16 drugs to prevent DNR/pNCD, and only 3 of them were shown to be associated with benefits: lidocaine, magnesium sulfate, and ketamine [43]. In the original studies, lidocaine and magnesium sulfate were administered intra- and postoperatively, while ketamine was tested as a single dose during induction of general anesthesia [45,46,47,48]. The non-pharmacological tested approach includes environmental adaptations (such as normal circadian function and good sleep quality), behavioral interventions, intraoperative depth of anesthesia monitoring with the bispectral index (BIS) or cerebral oxymetry, postoperative rehabilitation, psychological and social supports, and complementary and alternative medicine [8].

Clinical use of insulin as non-glycemia management therapy administered intravenously was firstly described for morphine addiction treatment, schizophrenia symptom mitigation, and dementia praecox. Hypoglycemic shock induced by insulin was shown to have a psychotropic effect in these patients. This approach consisted of four phases (preparatory phase, shock phase, rest phase, and terminal phase) and was abandoned after the introduction of antipsychotic drugs. Subsequently, the administration of intranasal insulin was found to be safe and have positive effects on neurocognitive performance, memory performance, daily activity, and brain volume during acute, subacute, and chronic phases after ischemic stroke events, both in healthy individuals and in patients with memory impairment such as MCI, AD, PD, and multiple system atrophy. Several approaches have been tested to prevent DNR/pNCD, and these include pre-rehabilitation and enhanced recovery. There are no effective pharmacological therapies that have reached an adequate level of evidence to warrant clinical use, and intranasal insulin might represent an innovative approach [13,49,50]. Of interest, when administered intranasally, insulin bypasses the BBB and reaches the brain along perineural spaces of the olfactory and trigeminal nerves [33,49]. Subsequently it is distributed along cerebral perivascular spaces without raising peripheral insulin levels or lowering blood glucose. This might explain the absence of associated effects on systemic glycemia, thus making this therapy suitable for perioperative use with no relevant effects on the blood glucose concentration.

The main limitations of the narrative review consist of the limited clinical evidence in the current literature of the causative role of anesthesia exposure in cognitive impairment >6 months postoperatively and the role of intranasal insulin administration in preventing the onset of DNR/pNCD. Another limitation is the lack of ultimate indications of the usefulness and appropriateness of nasal delivery systems for insulin administration. A recent clinical trial on patients with AD reported no differences in the use of two different tools for intranasal insulin administration [19]. This study could be used to design clinical trials in the future.

## 5. Future Perspectives

The promising role of the potential effects of intranasal insulin administration in mitigating or possibly avoiding the onset of DNR/pNCD and behavioral impairment after general anesthesia should stimulate researchers to design clinical trials aimed at confirming or excluding these findings in human patients. Since the therapeutic effects of intranasal insulin administration have been reported in different clinical settings, including healthy individuals, patients with acute ischemic stroke, and patients with memory impairment with different etiology and severity, there is room to test its effects also in a perioperative setting. Ideally, different population subsets should be tested in specifically designed RCTs, including healthy patients and individuals with prior cognitive deficits admitted for scheduled surgery and randomized to receive either intranasal insulin or saline. Among the relevant outcomes that should be investigated, there is the cognitive status before and after surgery, possibly with long-term follow-up.

## 6. Conclusions

DNR/pNCD are major complications that can occur after surgery and anesthesia. Several pharmacological and non-pharmacological strategies have been tested to prevent their onset, but few prove to be effective. The use of intranasal insulin, considering the available preclinical trials and the limited clinical evidence, has the potential to effectively contribute to the prevention of DNR/pNCD. This therapeutic effect can be explained through the action on insulin brain receptors and interference with molecular mechanisms of anesthesia-induced cognitive decline. Moreover, the possibility that intranasal administration of insulin could represent a preemptive treatment unfolds very important issues that need to be explored. Further confirmation of the molecular basis of this insulin-related cognition-sparing effect could both strengthen the evidence collected thus far and represent a solid therapeutic target. Future clinical studies should be appropriately designed—with selected patient population, preoperative screening, and postoperative long-term follow up—to further confirm available evidence on the use of intranasal insulin administration perioperatively to reduce or prevent the incidence of DNR/pNCD after anesthesia.

## Figures and Tables

**Table 1:** Preclinical evidence: intranasal insulin for preventing changes in the brain molecular pattern and/or neurobehavioral after anesthesia exposure in animal models.

Reference	Results
Chen, Y., et al. [20]	Intranasal insulin administered before anesthesia exposure in AD mice was associated with lower phosphorylation levels of tau protein.
Zhang, Y., et al. [21]	Intranasal insulin administered before anesthesia exposure in wild-type mice was shown to reduce hyperphosphorylation of tau proteins and to prevent spatial learning impairment.
Chen, Y., et al. [22]	Intranasal insulin administration in AD mice before anesthesia exposure was shown to prevent anesthesia-induced spatial learning and memory deficit and long-term neurobehavioral changes.
Li, H., et al. [23]	Intranasal insulin administered in neonatal mice before anesthesia exposure was reported to prevent long-term behavioral abnormalities, changes in synaptic proteins, and neuronal apoptosis.
Dai, C.L., et al. [24]	Intranasal insulin administration before anesthesia exposure in neonatal mice was shown to prevent anesthesia-related behavioral and cognitive impairment associated with specific brain areas such as the amygdala, hippocampus, frontal cortex, and cingulate cortex.
Yu, Q., et al. [25]	Intranasal insulin administered before anesthesia exposure in wild-type aged mice was demonstrated to promote mTOR-eEF2 signaling pathway and prevent anesthesia-induced reduction in brain synaptic proteins and the BDNF and cognitive decline.
Li, X., et al. [26]	Intranasal insulin administration in aged mice exposed to anesthesia was shown to prevent hyperphosphorylation of tau proteins, loss of specific synaptic proteins, and cognitive defects.

AD, Alzheimer’s disease; BDNF, brain-derived neurotrophic factor; eEF2, eukaryotic elongation factor 2 kinase; mTOR, mammalian target of rapamycin-eukaryotic elongation factor 2.

**Table 2:** Clinical evidence: systemic, neurocognitive, and neuromolecular changes after insulin exposure.

Reference	Type of Study	Other Information	Results
Banting, F.G., et al. [27]	Case control	14-year-old boy with glycosuria, weight loss, increased frequency of day and night micturition	Extracts of insular tissue of the pancreas injected subcutaneously or intravenously led to reduction in the blood glucose concentration, abolishment of glycosuria, disappearance of acetone bodies from the urine, and increase in the of carbohydrates.
Sakel, M. [28]	Case series	Psychiatric patients under insulin-induced hypoglycemia/coma	Patients with morphine addiction and schizophrenia had a reduction in or the disappearance of hallucinations during insulin-induced hypoglycemia and a protraction of the lucid phase.
Mack, C.W., et al. [29]	Case series	19 dementia praecox patients undergoing insulin-induced hypoglycemic shock	Hypoglycemia shock induced by insulin has shown clinical improvement in dementia praecox patients.
Fink, M., et al. [30]	Randomized control trial	60 psychiatric patients: 30 patients received chlorpromazine by mouth with a median of 800 mg daily for at least 3 months; insulin coma induced in each of the other 30 patients	Chlorpromazine was safer, easier to administer, and suited to long-term management than an insulin coma in schizophrenic patients.
Begg, D.P. [31]; Dorn, A., et al. [32]	Chapter; observational study	Insulin transport into the brain/CSF; brain insulin production	Plasma insulin is transported through endothelial BBB cells by an IR-mediated transcytosis mechanism, while the hypothalamus, hippocampus, and brain stem produce insulin independently.
Lioutas, V.A. [33]	Review	Intranasal insulin administration in healthy, MCI, and AD participants	Intranasal insulin reaches the CNS through olfactory and trigeminal neurons without causing systemic hypoglycemia or hepatic first-pass metabolism; it increased the feeling of well-being and self-confidence and decreased anger in healthy participants, while in MCI/AD patients, it improved functional status and in AD patients general cognition performance.
Lioutas, V.A., et al. [34]	Perspective article	Role of intranasal insulin as a neuroprotective agent in ischemic stroke patients	Effects of intranasal insulin as a neuroprotective agent: suppression of pro-inflammatory transcription response, vasodilatory effects, induction of thrombolysis, reduction in infarct volume, anti-apoptotic effects, promotion of neurite regeneration, neurotransmission, and functional connectivity.
Benedict, C., et al. [35]	Randomized controlled trial	38 healthy participants; each group *n* = 19; intranasal insulin administration (4X40 UI/d) vs. placebo	Intranasal insulin administration in individuals without memory impairment was shown to improve recall ability and self-confidence in cognitive tasks after 8-week treatment.
Shemesh, E., et al. [36]	Systematic review	8 studies: healthy participants, MCI, or AD patients	High doses of intranasal insulin (160 IU/die) compared to low doses (≥60 IU/die) in healthy individuals were shown to induce beneficial effects; in MCI/AD patients, long-term intranasal insulin administration of 20 UI showed a beneficial effect on cognitive functions.
Reger, M.A., et al. [37]	Randomized controlled trial	35 healthy adults and 26 MCI/AD patients receiving saline, 20 IU, or 40 IU intranasal insulin administration	Intranasal insulin administration in APOE4- MCI/AD patients was shown to facilitate recall of verbal memory, with stronger effects compared to APOE4+ MCI/AD patients and healthy adults.
Avgerinos, K.I., et al. [38]	Systematic review	7 studies of 293 MCI/AD patients receiving intranasal insulin administration	Improved story recall performance in APOE4- MCI/AD patients.
Benedict, C., et al. [39]	Review	Intranasal insulin administration in healthy individuals or AD patients	Memory-improving effect of the CNS in both healthy and AD individuals.
Chapman, C.D., et al. [40]	Review	Cognitively healthy or MCI/AD individuals treated with intranasal insulin	Cognitive and functional status improvement and preservation of brain volume.
Claxton, A., et al. [41]	Randomized controlled trial	60 MCI/AD patients receiving intranasal placebo, 20 IU, or 40 IU of insulin detemir for 21 days	40 IU intranasal detemir improved verbal memory in MCI/AD APOE-4 allele carrier patients and visuospatial and verbal working memory for all participants.
Novak, P., et al. [42]	Randomized controlled trial	16 PD/MSA patients receiving placebo or 40 IU insulin intranasally	Intranasal insulin administration improved cognitive and functional performance in PD patients, while in MSA patients, it induced a lack of disease progression.

AD, Alzheimer’s disease; APOE, apolipoprotein E; BBB, blood–brain barrier; CNS, central nervous system; CSF, cerebrospinal fluid; IRs, insulin receptors; IU, international unit; MCI, mild cognitive impairment; MSA, multiple system atrophy; PD, Parkinson’s disease.

## Data Availability

Data is contained within the article.
